# Cardiac recovery from pressure overload is not altered by thyroid hormone status in old mice

**DOI:** 10.3389/fendo.2024.1339741

**Published:** 2024-02-22

**Authors:** Helena Kerp, Janina Gassen, Susanne Camilla Grund, Georg Sebastian Hönes, Stefanie Dörr, Jens Mittag, Nina Härting, Frank Kaiser, Lars Christian Moeller, Kristina Lorenz, Dagmar Führer

**Affiliations:** ^1^ Department of Endocrinology, Diabetes and Metabolism, University Hospital Essen, University of Duisburg-Essen, Essen, Germany; ^2^ Cardiovascular Pharmacology, Leibniz-Institut für Analytische Wissenschaften-ISAS-e.V., Dortmund, Germany; ^3^ Institute of Endocrinology and Diabetes and Center for Brain, Behavior and Metabolism, University Hospital Schleswig-Holstein (UKSH), University of Lübeck, Lübeck, Germany; ^4^ Institute of Human Genetics, University Hospital Essen, University of Duisburg-Essen, Essen, Germany; ^5^ Institute of Pharmacology and Toxicology, University of Würzburg, Würzburg, Germany

**Keywords:** maladaptive cardiac hypertrophy, thyroid hormone, transverse aortic constriction, aging, thyroid hormone receptor, deiodinase, pressure overload, heart failure

## Abstract

**Introduction:**

Thyroid hormones (THs) are known to have various effects on the cardiovascular system. However, the impact of TH levels on preexisting cardiac diseases is still unclear. Pressure overload due to arterial hypertension or aortic stenosis and aging are major risk factors for the development of structural and functional abnormalities and subsequent heart failure. Here, we assessed the sensitivity to altered TH levels in aged mice with maladaptive cardiac hypertrophy and cardiac dysfunction induced by transverse aortic constriction (TAC).

**Methods:**

Mice at the age of 12 months underwent TAC and received T4 or anti-thyroid medication in drinking water over the course of 4 weeks after induction of left ventricular pressure overload.

**Results:**

T4 excess or deprivation in older mice had no or only very little impact on cardiac function (fractional shortening), cardiac remodeling (cardiac wall thickness, heart weight, cardiomyocyte size, apoptosis, and interstitial fibrosis), and mortality. This is surprising because T4 excess or deprivation had significantly changed the outcome after TAC in young 8-week-old mice. Comparing the gene expression of deiodinases (Dio) 2 and 3 and TH receptor alpha (TRα) 1 and the dominant-negative acting isoform TRα2 between young and aged mice revealed that aged mice exhibited a higher expression of TRα2 and Dio3, while expression of Dio2 was reduced compared with young mice. These changes in Dio2 and 3 expressions might lead to reduced TH availability in the hearts of 12-month-old mice accompanied by reduced TRα action due to higher TRα2.

**Discussion:**

In summary, our study shows that low and high TH availability have little impact on cardiac function and remodeling in older mice with preexisting pressure-induced cardiac damage. This observation seems to be the result of an altered expression of deiodinases and TRα isoforms, thus suggesting that even though cardiovascular risk is increasing with age, the response to TH stress may be dampened in certain conditions.

## 1 Introduction

Cardiovascular diseases are still the leading cause of death (1). Several clinical and preclinical studies suggest a close association of thyroid dysfunction with cardiovascular diseases (2–7). In this context, systemic hyper- and hypothyroidism have been revealed as risk factors for heart failure (8). Aging acts as an important further modifier, as the prevalence of both thyroid dysfunction and cardiac diseases increases with age (9–11). However, the relevance and interplay of altered thyroid hormone (TH) availability, age, and their impact on the progression or recovery of cardiac diseases are still not fully understood.

THs increase the heart rate and cardiac inotropy and decrease the arterial vascular smooth muscle tone leading to reduced cardiac afterload (3, 12–14). These TH effects involve the regulation of sarcomeric proteins, e.g., myosin heavy chain alpha and beta, proteins involved in the regulation of calcium homeostasis, e.g., the sarcoplasmic/endoplasmic reticulum Ca(2+)ATPase 2a2 and the ryanodine receptor 2 (15–17). Administered for a certain duration, THs have shown to reduce cardiac afterload, improve cardiac output, and lead to a so-called physiological, compensatory cardiac hypertrophy (3, 18–24).

In our previous study in 8-week-old mice, we found a low TH state to be beneficial under conditions of left ventricular pressure overload induced by transverse aortic constriction (TAC). Reduced cardiac hypertrophy and improved cardiac function were evident after 4 weeks of TH deprivation. In contrast, TH excess was not beneficial in this situation as it did not prevent cardiac hypertrophy, increased apoptosis, and had a negative impact on cardiac function (25). Under these conditions, TH excess led to a so-called pathological cardiac hypertrophy accompanied by increased interstitial fibrosis and cardiomyocyte apoptosis (26, 27).

Here, we aimed to investigate whether or to what extent older mice at the age of 12 months develop changes in heart function and morphology after TAC in response to TH deprivation or high-dose TH availability. In line with Kerp et al. (25), modulation of thyroid status was started after 1 week of TAC when cardiac hypertrophy and the first signs of cardiac depression have developed. In contrast to our previous study in young mice, low and high TH availability showed little impact on cardiac hypertrophy, apoptosis, fibrosis, or cardiac function. Our results suggest that these age-related differences are associated with altered gene expression levels of TRα isoforms and deiodinases 2 and 3 in 12-month-old mice compared with 8-week-old mice. Therefore, we suggest that even though cardiovascular risk is increasing with age, the response to TH stress may be dampened.

## 2 Materials and methods

### 2.1 Animals

Male wild-type C57BL/6JRj mice (Janvier Labs, France) were studied at the age of 12 months as previously described (25). Briefly, all mice were single-housed under standard conditions (room temperature 23°C ± 1°C; humidity 55% ± 10%; 12:12 h light–dark cycle). Standard chow diet and water were provided *ad libitum*. All animal experiments were performed in accordance with the German Regulations for Laboratory Animal Science (GV-SOLAS) and the European Health Law of the Federation of Laboratory Animal Science Associations (FELASA). The experimental protocols were approved by the Landesamt für Natur, Umwelt und Verbraucherschutz Nordrhein-Westfalen, Germany (LANUV-NRW, AZ 84-02.04.2016.A261).

### 2.2 Transverse aortic constriction

**Figure 1:**
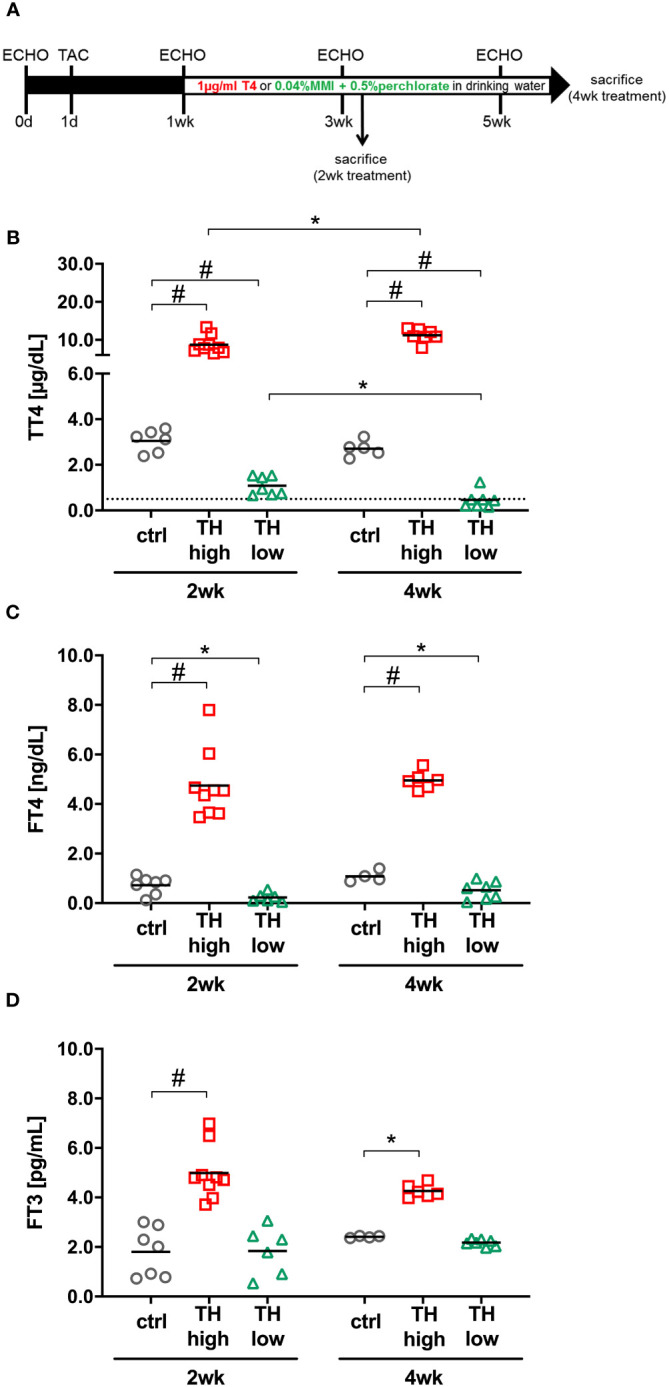
Study design and serum thyroid hormone (TH) status. Twelve-month-old male C57Bl/6 mice were subjected to transaortic constriction (TAC) at experimental day 1. Echocardiographic measurements (ECHO) were conducted 1 day prior to TAC (day 0) and after 1, 3, and 5 weeks. One week after TAC, oral T4 treatment (TH high) and TH deprivation [MMI/ClO_4_^−^ in combination with low iodine diet (TH low)] were started. Controls (ctrl) received drinking water without supplements. Mice were sacrificed 2 or 4 weeks after induction of TH dysfunction **(A)**. TT4 **(B)**, FT4 **(C)**, and FT3 **(D)** serum concentrations confirmed TH excess and TH deprivation at both time points in the two treatment groups compared with controls **(C)**. The dotted line represents the detection limit of the assay and the values below were calculated from the standard curve. *p < 0.05, ^#^ *p* < 0.0001 by two-way ANOVA and Tukey’s *post-hoc* analysis; ctrl, control; wk, weeks.

Mice were subjected to TAC using a 25-gauge needle to induce chronic left ventricular pressure overload as previously described (28, 29). One day prior to TAC and 1, 3, and 5 weeks after TAC, echocardiography was performed (**Figure 1A**). Mice with an aortic pressure gradient below 60 mmHg at 1 week after TAC were excluded from the study.

### 2.3 Echocardiography

Transthoracic echocardiograms were performed in a blinded fashion using the Vevo 3100 high-resolution imaging system (FUJIFILM VisualSonics, Amsterdam, The Netherlands) and the MX550D transducer with pentobarbital (20–35 mg/kg body weight, i.p.) as an anesthetic. Two-dimensional M-mode images in the short axis view at the proximal level of the papillary muscles were used to evaluate end-diastolic and end-systolic intraventricular septal (IVSd/IVSs), left ventricular posterior wall thickness (LVPWd/LVPWs), and end-diastolic and end-systolic left ventricular internal diameters (LVIDd/LVIDs). Pulsed-wave Doppler measurements were performed to evaluate the peak blood flow velocities at the site of constriction (*V*_max_ (mm/s)). Fractional shortening (FS) and aortic pressure gradients (mmHg) were calculated using the VisualSonics Cardiac Measurements software. The data represent the average of at least six cardiac cycles. The investigators were blinded regarding treatment groups during measurements and data analysis. Measurements at heart rates below 450 bpm were excluded.

### 2.4 Treatment

Mice were subjected to two different treatment protocols: 1) to increase TH availability (TH high), mice received thyroxine (T4) in drinking water [1 μg/ml, Sigma-Aldrich (T2376), USA; stock solution: 100 µg/ml of T4 solved in 40 mM of NaOH and 2 g/L of bovine serum albumin] (30). 2) For TH deprivation (TH low), mice received a low-iodine diet (LoI; MD.1571, Envigo, USA) and drinking water supplemented with 0.04% methimazole [MMI, Sigma-Aldrich (301507), USA], 0.5% sodium perchlorate [ClO_4_^−^, Sigma-Aldrich (310514), USA], and 0.3% saccharine as sweetener [Sigma-Aldrich (240931), USA) (LoI/MMI/ClO_4_^−^]. In the control group (ctrl), animals were fed a normal diet (MD.1572, Envigo, USA) and received drinking water without supplements. Using the above-described treatment protocol, we have extensively studied male mice without TAC surgery under chronic high or low TH availability and, therefore, decided to investigate male mice in order to focus on the impact of age and TH sensitivity in pathological cardiac hypertrophy (31–33). Furthermore, TAC sham operation has previously been shown not to affect parameters of cardiac function or morphology (28, 29, 34). For these reasons and in line with the “3R concept” in animal research, the present study was done without including an additional sham-operated control group. Except for the low iodine content in the TH deprivation group, the caloric and nutritional composition of the diet was comparable for all mice (MD.1572, Envigo, USA, for the control and T4-treated mice). The indicated treatment started 1 week after TAC surgery and was continued for 2 or 4 weeks (**Figure 1A**).

### 2.5 Organ isolation and serum measurements

For organ extraction, mice were anesthetized by i.p. injection of 200 µl of ketamine/xylazine mixture [150 µl of 100 mg/ml ketamine (Betapharm, Germany) and 50 µl of 20 mg/ml xylazine (Ceva, Germany)], and blood was withdrawn by cardiac puncture. Mice were perfused with heparinized saline by transcardial perfusion. Hearts were shock-frozen in liquid N_2_ and stored at −80°C or fixed in 4% buffered formalin (Formafix, Germany). Blood samples were placed on ice for 30 min and centrifuged, and the concentrations of free triiodothyronine (FT3), free T4 (FT4), and total T4 (TT4) in mouse sera were measured according to the manufacturer’s instructions of commercial ELISA kits (DRG Instruments GmbH, Marburg, Germany) as previously described (25, 31, 35). The detection limit was 0.5 µg/dl, 0.05 ng/dl, and 0.05 pg/ml for TT4, FT4, and FT3, respectively. Values were calculated from the standard curve.

### 2.6 RNA isolation and qRT-PCR

Total cardiac RNA was isolated and reverse-transcribed to cDNA as previously described (35). In compliance with the guidelines for RT-PCR of MIQE, we used three reference genes to assure correct normalization and calculation: *Gapdh* (glyceraldehyde-3-phosphate dehydrogenase), *Rn18s* (18S ribosomal RNA), and *Polr2a* (polymerase RNA II). The primer sequences are listed in **Supplementary Table 1**. Analysis and calculation of the fold changes of gene expression were applied to Ct values ≤35. In addition to the 12-month-old mice, RNA samples of the analogously treated 8-week-old mice (generated and published in Kerp et al. (25)) were used for some gene expression analyses as indicated.

### 2.7 Histological staining

Formalin-fixed hearts were embedded in paraffin. Five-micrometer sections were used for staining. Hematoxylin and eosin (H&E) staining was used for the determination of cardiomyocyte size, and Sirius Red was used for the analysis of fibrosis as described previously (28). For quantification, stained sections were imaged using an Olympus BX51 microscope (Olympus Life Science, Tokyo, Japan). Cross-sectional areas of cardiomyocytes were determined (*n* = 37–88 cells per animal) using ImageJ. Only cells with a central nucleus were included in the analysis. Fibrosis was calculated as the ratio of red-stained/myocardial area via Adobe Photoshop. Analysis and quantification were performed by blinded researchers.

### 2.8 TUNEL assay

After dewaxing and rehydration, cardiac sections were permeabilized using proteinase K. For TUNEL (terminal deoxynucleotidyl transferase dUTP nick-end labeling) staining, an *in-situ* detection kit was used according to the manufacturer’s protocol (Sigma-Aldrich, USA). Sample pretreatment with DNase I served as positive control and TUNEL reaction mixture without terminal transferase (TdT) served as negative control. DAPI (D1306, Thermo Fisher Scientific, USA) and wheat germ agglutinin (W11261, Thermo Fisher Scientific, USA) were used as counterstains for cell nuclei and membranes. Olympus BX51 upright microscope was used for the imaging, and quantification was conducted in a blinded manner.

### 2.9 Statistical analysis

GraphPad Prism 6 software was used for statistical analysis. Data were checked for normality prior to statistical testing. Two-way ANOVA considering the treatment group (ctrl, TH high, TH low) and time points (regarding weeks after TAC or TH modulation, respectively) followed by Tukey’s *post-hoc* analysis was used. *p*-values of *p < 0.05, **p < 0.01, ***p < 0.001, and ^#^ *p* < 0.0001 were considered significant. Outliers were identified using the GraphPad outlier test (excluded if *p* < 0.05).

## 3 Results

TH availability may alter the progression of heart disease and the development of heart failure (2, 5, 36). To investigate the impact of aging on this process, we subjected 12-month-old male mice to chronic left ventricular pressure overload induced by TAC. This intervention leads to pathological growth of the heart, i.e., maladaptive cardiac hypertrophy accompanied by interstitial fibrosis and cardiomyocyte death and subsequently to decreased fractional shortening (FS) and the development of congestive heart failure (34).

Changes in TH availability were induced by T4 administration or anti-thyroidal drug treatment (LoI/MMI/ClO_4_^−^), respectively, initiated 1 week after TAC surgery and continued over 4 weeks as depicted in the study design (**Figure 1A**). As expected, 2 and 4 weeks of T4 treatment resulted in increased TT4, FT4, and FT3 serum concentrations (**Figures 1B–D**). LoI/MMI/ClO_4_^−^ treatment significantly decreased TT4 and FT4 but not FT3 serum concentrations (**Figures 1B–D**).

### 3.1 TH effects on maladaptive cardiac hypertrophy are reduced in 12-month-old mice

**Figure 2:**
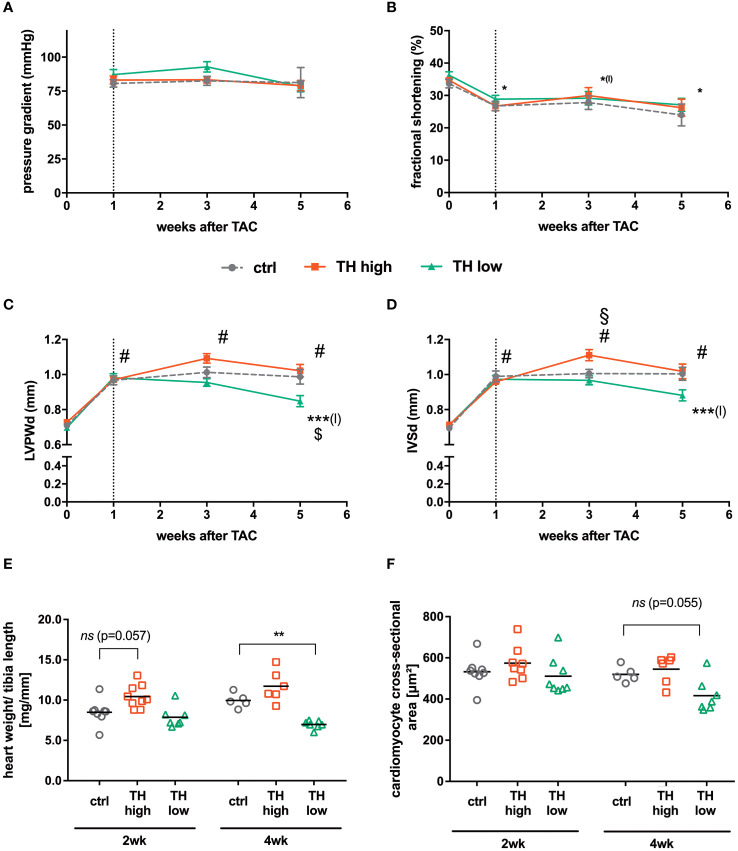
Cardiac function and hypertrophy characteristics. Echocardiography was conducted before TAC and 1, 3, and 5 weeks after surgery. After 1 week, pressure gradients confirmed successful TAC surgery **(A)** and fractional shortening decreased in all indicated groups compared with baseline values without an apparent impact on thyroid status **(B)**. For cardiac hypertrophy analysis, diastolic left ventricular posterior wall (LVPWd; **C**) and interventricular septum (IVSd; **D**) thickness were calculated. In addition, heart weight to tibia length ratios **(E)** and cardiomyocyte cross-sectional areas **(F)** were assessed 3 or 5 weeks after TAC [i.e., after 2 or 4 weeks of subsequent modulation of TH status by T4 treatment (TH high) or TH deprivation (TH low)]. TH deprivation reduced cardiac hypertrophy parameters (LVPWd, IVSd, and heart weight to tibia length). Values are indicated as mean ± SEM; for *n*, see **Supplementary Table 1A**. ^#^ *p* < 0.0001 (for echocardiographic parameters all groups vs. basal), *^(l)^ *p* < 0.05 (for TH high vs. basal), ***^(l)^ *p* < 0.001 (TH low vs. baseline after TAC), $*p* < 0.05 (TH low vs. ctrl at 5 weeks after TAC), and §*p* < 0.05 (TH high vs. ctrl at 3 weeks after TAC) by two-way ANOVA and Tukey’s *post-hoc* analysis; ctrl, control; wk, weeks. The dotted line represents the start of T4 or LoI/MMI/ClO_4_^−^ treatment.

Serial echocardiographic measurements were performed to monitor the progression of cardiac hypertrophy and cardiac dysfunction in response to TAC. Aortic pressure gradients induced by TAC were comparable in the different treatment groups and were stable throughout the experiment (**Figure 2A**). At 1 week of TAC and before manipulation of TH status commenced, a significantly decreased FS was confirmed. Of note, the subsequent alteration of TH levels, i.e., gradual TH increase and TH deprivation, did not alter FS over 4 weeks of treatment (**Figure 2B**; **Table 1**). The thickness of the left ventricular wall was significantly increased after 1 week of TAC as depicted by both posterior wall and septum diameters compared with baseline values and hardly changed during the following 4 weeks of observation in the euthyroid and T4-treated groups. Only in the TH low group, a significant decrease of the left ventricular posterior wall thickness (LVPWd; **Figure 2C**) and of the interventricular septum thickness (IVSd; **Figure 2D**) was observed after 4 weeks of TH deprivation. However, the left ventricular inner diameter was unaltered at all time points and in all treatment and control groups (**Supplementary Figure 1A**).

**Table 1:** Reduction of FS over time in 12-month-old compared with 8-week-old groups (23), receiving the same experimental conditions of TAC surgery and modulation of TH status (TH high: T4 treatment and TH low: TH deprivation).

	8 weeks old	12 months old
1 week after TAC vs. basal	20.7% ± 2.40%	18.8% ± 2.76%
5 weeks after TAC vs. basal (control)	33.9% ± 4.69%	33.4% ± 10.27%
5 weeks after TAC vs. basal (TH high)	46.2% ± 3.51%	27.8% ± 6.82%
5 weeks after TAC vs. basal (TH low)	19.7% ± 3.65%	28.0% ± 6.72%

These echocardiographic data on cardiac hypertrophy were validated by heart weight to tibia length ratios and cardiomyocyte cross-sectional area. Here, a significantly reduced heart weight to tibia length ratio was found under TH deprivation (**Figure 2E**), and a similar trend was observed for the cardiomyocyte cross-sectional area (**Figure 2F**). No significant differences were detected after 2 and 4 weeks of T4 treatment (**Figures 2E, F**). Thus, elevated TH serum concentrations did not affect the development of cardiac hypertrophy, whereas low TH serum concentrations had a mild impact on the progression of cardiac hypertrophy. Neither cardiac function nor lung weight as a measure of cardiac congestion was influenced by altered TH levels during the 4-week treatment interval after induction of left ventricular pressure overload (**Supplementary Figure 1B**).

### 3.2 TH-dependent development of fibrosis and apoptosis is attenuated after TAC in 12-month-old mice

**Figure 3:**
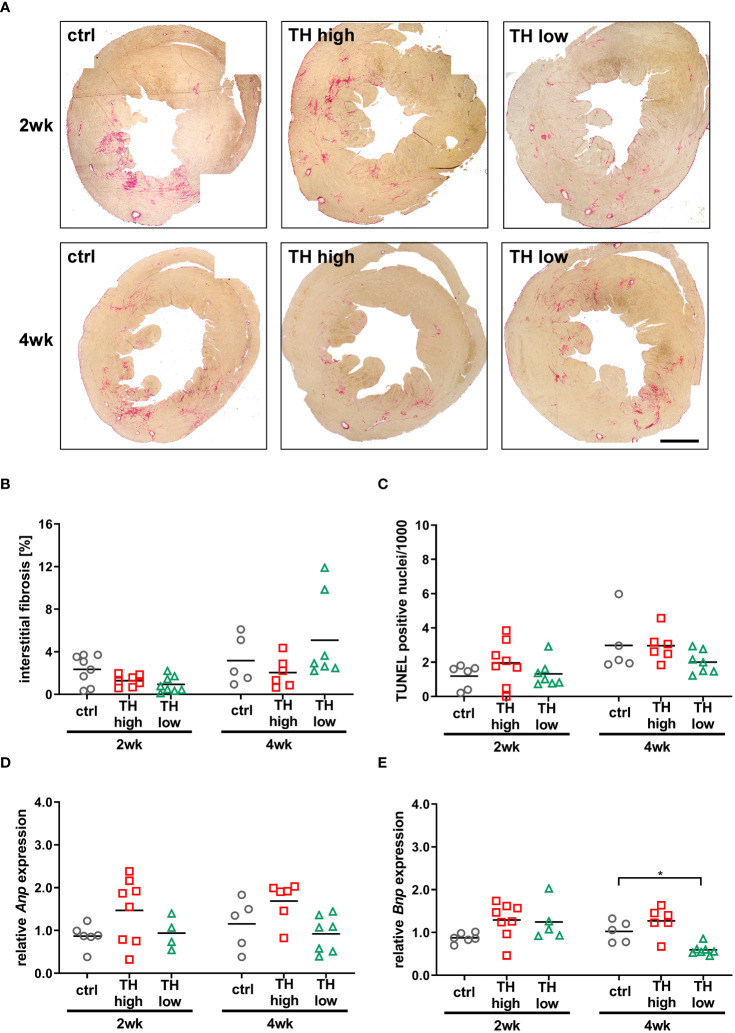
Analysis of fibrosis, apoptosis, and cardiac stress markers at 3 or 5 weeks after TAC [i.e., after 2 or 4 weeks of subsequent modulation of TH status by T4 treatment (TH high) or TH deprivation (TH low)]. Representative pictures of Sirius Red-stained sections (**A**, scale bar = 1,000 µm) and relative quantification of interstitial fibrosis **(B)** are shown. TUNEL-positive nuclei were counted to assess apoptotic rate **(C)** and *Anp* and *Bnp* expression as cardiac stress markers **(D, E)**. TH deprivation reduced cardiac *Bnp* expression in 12-month-old mouse hearts at 5 weeks after TAC. Scatter dot plot and mean in all panels, *p < 0.05 by two-way ANOVA and Tukey’s *post-hoc* analysis; ctrl, control; wk, weeks.

Apart from cardiac hypertrophy, deposition of collagen contributing to fibrosis and cardiomyocyte apoptosis are key features of pathological cardiac remodeling, which leads to cardiac dysfunction, altered electrophysiological dynamics, and cardiac tissue stiffness (37). Interstitial fibrosis was histologically analyzed by Sirius Red staining and apoptotic cell death by TUNEL staining (29). No significant alterations in the extent of fibrosis (**Figures 3A, B**) and apoptosis (**Figure 3C**) were evident between the treatment groups after 2 and 4 weeks. Furthermore, cardiac expression of atrial natriuretic peptide (*Anp*) and brain natriuretic peptide (*Bnp*), markers that correlate with the development of heart failure and pathological cardiac hypertrophy, was analyzed by qRT-PCR (38, 39). *Anp* expression levels did not change in response to altered TH status, while cardiac *Bnp* expression was significantly lowered after 4 weeks of TH deprivation compared with controls (**Figures 3D, E**).

Moreover, despite the absence of apparent features of heart failure, three death events occurred in the control group and two in the T4 treatment group in comparison to none in TH-deprived mice within 4 weeks of modulation of TH status.

### 3.3 TH responsiveness of TAC-subjected hearts reflects low and high TH tissue states

**Figure 4:**
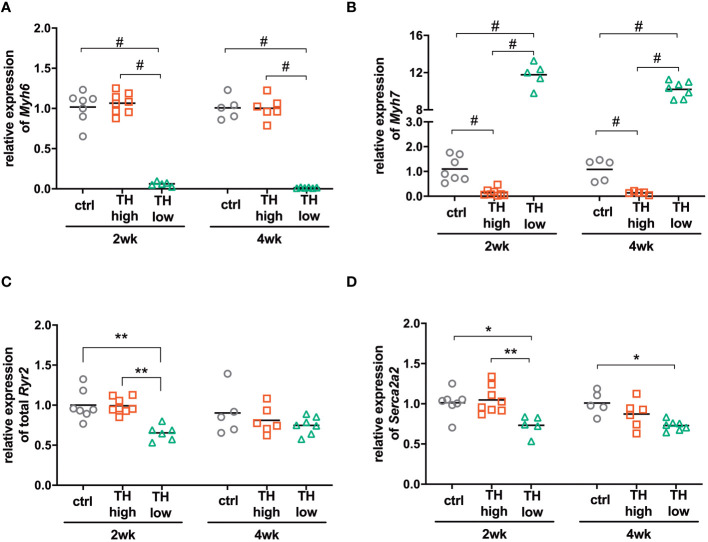
Expression of TH-responsive genes in the hearts of mice subjected to TAC and subsequent modulation of TH status by T4 treatment (TH high) or TH deprivation (TH low). The amount of *Myh6* **(A)**, *Myh7* **(B)**, *Ryr2* **(C)**, and *Atp2a2* **(D)** transcripts was determined in mouse hearts by qRT-PCR after 2 and 4 weeks of treatment. Scatter dot plot and mean in all panels, one-way ANOVA with Tukey’s *post-hoc* test, *p < 0.05, **p < 0.01, ^#^ *p* < 0.0001; ctrl, control; wk, weeks.

To answer whether the higher or lower TH availability in mice translated into altered expression of genes associated with hypo- or hyperthyroidism (13) and TH target gene expression of myosin heavy chain alpha (*Myh6*) and beta (*Myh7*), ATPase sarcoplasmic/endoplasmic reticulum Ca2+ transporting 2 (*Serca2a2*) and ryanodine receptor 2 (*Ryr2*) was analyzed in mouse hearts, sacrificed after 2 and 4 weeks of treatment. As observed upon TH deprivation in previous studies (25), *Myh6* levels were significantly lowered and *Myh7* expression increased after 2 and 4 weeks of low TH treatment. High TH treatment did not affect *Myh6* expression but resulted in reduced expression of *Myh7* (**Figures 4A, B**). Expression of *Ryr2* was decreased in the low TH group only after 2 weeks of treatment, whereas expression of *Serca2a2* was significantly reduced after 2 and 4 weeks under TH deprivation (**Figures 4C, D**). Thus, both treatments leading to high or low TH serum concentration, respectively, caused the expected gene alteration in cardiac muscle. However, the effect on cardiac TH target gene expression was greater under TH deprivation than under T4 treatment.

We also measured the expression of the TAC-associated genes (*Col1a1*, *Atp5g1*, *Ndufa5*, *Uqcrq*, *Mmp2*, and *Pdk4*) according to Li et al. (40). There were no considerable differences detectable except for the expression of *Pdk4* that showed an expression pattern that correlated with TH treatment (TH high and low, respectively) in the 4-week groups (**Supplementary Figure 2**).

### 3.4 Attenuated TH response in 12-month-old mice is associated with altered TRα isoform and deiodinase expression

**Figure 5:**
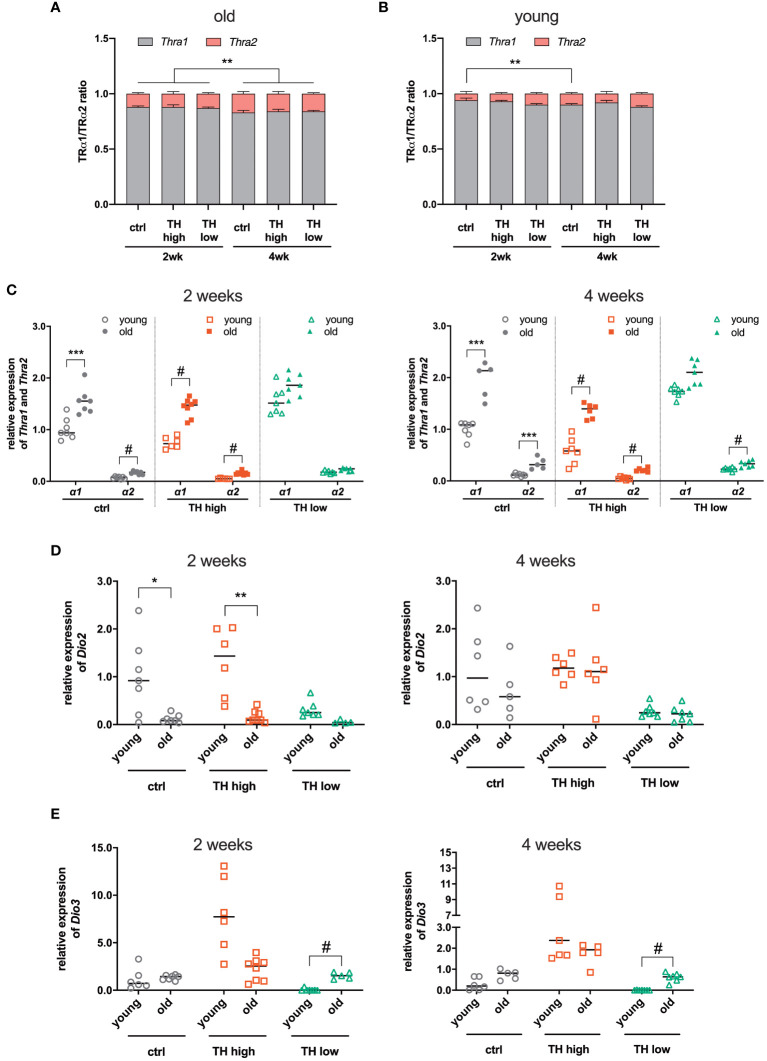
Comparison of gene expression between young and old mice after TAC. The expression ratio of TRα isoforms 1 and 2, encoded by *Thra1* and *Thra2* transcripts, respectively, in the hearts from **(A)** old (12-month-old) and **(B)** young mice (8-week-old) was assessed by qRT-PCR. **(C)** Relative gene expression of TRα1 and 2 in the hearts from young and old mice, 2 and 4 weeks after treatment, as well as *Dio2* **(D)** and *Dio3* **(E)**. Scatter dot plot and mean in all **(C, D)**, one-way ANOVA with Tukey’s *post-hoc* test, *p < 0.05, **p < 0.01, ***p < 0.001, ^#^ *p* < 0.0001; ctrl, control; wk, weeks.

Compared with young TAC mice (8-week-old), as previously published (25), we noticed reduced TH effects on TAC-related parameters in this aged cohort. To identify the underlying molecular causes, we decided to analyze TH-signaling features, i.e., gene expression of deiodinases and TR isoforms. Gene expression analyses of *Thra* and *Thrb* encoding for TH receptor alpha (TRα) and beta (TRβ), respectively, confirmed the predominant expression of TRα over TRβ in the heart (**Supplementary Figure 3**). Expression of TRα was decreased under high TH and increased under low TH, while TRβ was unaffected (**Supplementary Figure 3**). Interestingly, in older mice, the analysis of TRα isoform expression revealed that the proportion of TRα2, the isoform that is not able to bind TH, was increased compared with young mice. The expression of TRα2 further increased after 4 weeks of treatment, irrespective of the treatment regime (**Figure 5A**). This increase of the TRα2 isoform was less pronounced in young mice (**Figure 5B**). Moreover, the older mice had in general a higher proportion of TRα2 (2 weeks, 12.0% ± 1.3% vs. 7.7% ± 1.3% and 4 weeks 16.3% ± 1.7% vs. 10% ± 1.3%, old vs. young, respectively). A closer look at the TRα isoform distribution revealed that the expression of both TRα1 and TRα2 is higher in older mice compared with young mice (**Figure 5C**). Next, we measured the gene expression levels of deiodinases 2 and 3 encoded by *Dio2* and *Dio3*, respectively. After 2 weeks of treatment, the expression of *Dio2* was significantly higher in euthyroid and T4-treated young mice compared with the same groups of 12-month-old mice (**Figure 5D**, left panel). This difference disappeared after 4 weeks of treatment (**Figure 5D**, right panel). Interestingly, contrary to *Dio2*, the expression of *Dio3* was higher in euthyroid and hypothyroid older mice compared with young mice, reaching a significant difference under TH deprivation (**Figure 5E**).

## 4 Discussion

In our study, we investigated the impact of high and low TH availability after pressure overload-induced cardiac dysfunction in male 12-month-old mice. The aim of the study was to delineate the sensitivity to altered TH levels in aged mice in the context of “predamaged” hearts with left ventricular pressure overload.

Chronic pressure overload is a pathological condition that triggers the development of heart failure. Left ventricular pressure overload can be caused by arterial hypertension or aortic stenosis and TAC as a mouse model mimicking such a condition is widely used. TAC induces the development of cardiac hypertrophy, fibrosis, an increase in apoptosis, and eventually cardiac dilatation and congestive heart failure, characteristic features of heart disease induced by chronic pressure overload (28, 41).

Previously, we showed that modulation of the TH status 1 week after TAC surgery in young 8-week-old mice impressively impacted the outcome of cardiac recovery. In contrast to controls, TH deprivation in young mice stopped the progression of cardiac hypertrophy and cardiac dysfunction with mild improvement of FS, whereas elevated TH availability increased apoptosis, did not prevent hypertrophy, and decreased FS (25).

These results prompted us to add aging as another important modifier to this experimental setup since the prevalence of cardiac and TH-related diseases increases with age (10, 11) and sensitivity to TH and cardiovascular outcome changes with aging. TH action in aged mice has been well-documented and findings from our previous studies suggested decreased systemic effects in aged animals (31, 35). The heart as one of the main TH target organs was more likely to have a preserved TH action compared with other organs (35, 42). However, chronic TH excess in old mice led to a similar extent of cardiac hypertrophy, tachycardia, and regulation of TH-responsive gene expression as in young mice, whereas chronic TH deprivation resulted in an attenuated cardiac phenotype with less pronounced bradycardia and degree of gene regulation (31).

The effects of aging on cardiac hypertrophy and cardiac disease in animal models have been occasionally reported. Hypertrophic response of the left ventricle 4 weeks after ascending aortic banding in rats of 9 and 18 months of age showed a diminished degree of myocardial hypertrophy in old rats in terms of muscle weight, size of myocytes, and protein content (43–45). Mouse studies reported contradictory results of either worse response to chronic pressure overload and lower survival of old animals (46) or less left ventricular remodeling and lower mortality than young mice after TAC (47). However, so far, the impact of TH on TAC-induced heart disease has not been studied in the context of aging.

Due to the limited availability of aged TAC mouse models, our first aim was to identify similar conditions in 12-month-old as compared with 8-week-old mice for our setup so that aortic pressure gradients would not differ in older mice. This was achieved by using a bigger needle size (25G) during TAC, which resulted in a similar decline in FS within 1 week after surgery in aged compared with our previous study in young mice (25) and confirmed our approach to achieve age-independent conditions for TAC within the experimental time prior to TH alterations. Four weeks of T4 or LoI/MMI/ClO_4_^−^ treatment resulted in distinct effects on fractional shortening in both age groups, i.e., fractional shortening worsened in young mice with high TH levels and improved with low TH levels, whereas fractional shortening was hardly affected by low or high TH levels in old mice (**Table 1**).

Thus, TH alterations had no obvious detrimental or beneficial effects within the studied time frame in older mice. As no age effect was noted in the control group, we consider these findings unlikely to be a result of an inappropriate experimental setting.

Another influencing aspect might be that treatment time and dosage have to be age-adjusted to achieve comparable effects in younger and older individuals. Of note, the modulation of TH status in the present study led to similar changes in TH serum concentrations and cardiac gene expression as observed in young mice, further supporting comparable experimental conditions between the study in young mice and the herein-reported study in older mice (25). However, FT3 was not significantly reduced by LoI/MMI/ClO_4_^−^ treatment as seen in young mice. We previously showed that aging is associated with a low thyroid state resulting in lower basal FT3 concentrations in old mice compared with young mice (35). Therefore, the missing drop in FT3 could be a consequence of lower basal FT3 concentrations attenuating the effect of LoI/MMI/ClO_4_^−^ treatment.

Age has an effect on the progression of TH-induced physiological cardiac hypertrophy as early studies described a delayed development of cardiac hypertrophy in senescent mice upon thyroxine treatment. However, already after 9 days of treatment, no age differences were apparent anymore. In addition, different dosages were tested, and even with the lowest (1.7 µg/g BW), no age dependency of cardiac hypertrophy was noted (35, 48). In our present study and in response to TAC, however, the responsiveness of the heart to high or low serum TH levels was diminished compared with young mice (25). Aging per se reduces basal TH tissue content (35) and alters TH-dependent gene expression in the heart (42). Here, we show that there is also a different expression of TRα isoforms and deiodinases 2 and 3 in 12-month-old mice compared with young mice that have undergone TAC surgery. Noteworthy, we detected a shift in the TRα isoform ratio toward more TRα2 expression. TRα2 is incapable of binding TH and, thus, has a dominant-negative effect on TRα1 action (49). This increase was not affected by TH modulation, i.e., TH deprivation and TH treatment. However, as there was more TRα2 in the hearts of all groups after 4 weeks of treatment, we suggest that the amount of TRα2 correlates with the progression of cardiac hypertrophy. This is supported by previous studies that showed a shift from TRα1 to TRα2 expression in the human heart during heart failure (50, 51). Moreover, elevated TRα2 expression was found to attenuate TRα1-mediated hypertrophy in this condition (52). These findings suggest that the higher expression of TRα2 reduces TH response in 12-month-old mouse hypertrophied hearts. Interestingly, in addition to higher levels of TRα2, we found a reduced expression of *Dio2* in aged mice. Reduced expression of *Dio2* leads to less conversion of T4 to T3, consequently resulting in lower local T3 tissue content. This is further accompanied by higher cardiac expression of the TH-inactivating Dio3 in aged compared with young mice and could further reduce TH availability in aged hearts. This is in line with previous findings reporting lower T3 content in cardiac tissue of old mice (35). Briefly, the combination of a reduced TH availability caused by a reduction in Dio2 and higher expression of Dio3 together with an increased expression of the dominant-negative TRα2 together might provide a molecular explanation for the diminished TH response in aged TAC mice. Therefore, these factors might contribute to the outcome of TH modulation in the pathophysiology of heart disease.

TH deprivation has been studied less frequently. Thus, it remains speculative whether treatment time or dosage would affect the outcome of TAC in older mice. In addition, other studies suggested that cardiac hypertrophy significantly depends on indirect TH action in the central nervous system rather than direct TH effects on heart tissue (53, 54). Thus, the observed age difference could also arise from altered central TH rather than cardiac TH signaling in young vs. old mice with induced pressure overload (33).

Most clinical studies include patients with differing ages, sex, comorbidities, and different causes of cardiac dysfunction as well as common endpoints such as all-cause mortality or cardiovascular death in correlation with the thyroid status so that conclusions for therapeutic TH modulation are difficult (8, 55, 56). TH modulation may have different impacts in different subgroups of heart failure patients, e.g., in heart failure patients with or without atrial fibrillation (56) and in patients with congestive or dilative heart failure, with or without preexisting thyroid dysfunction. In line with these diverse factors that may impact the outcome of TH modulation, e.g., via altered Dio2/3 and TRα1/2 ratios and thus the availability of TH, also the timing and extent of TH modulation may be relevant (57–60).

The data of our current mouse study are focused and limited to TH sensitivity in 12-month-old mice after pressure overload-induced hypertrophy and cardiac dysfunction but suggest that TH modulation (high and low levels) at an older age—at least within a certain time frame and in the herein investigated male mice—has less impact to foster key risk factors of heart failure.

Taken together, certain subgroups of patients with preexisting cardiovascular disease may respond to manipulation of TH status with a beneficial or detrimental outcome. Our study suggests a different outcome to TH modulation based on age; thus, the recognition of differences due to, e.g., age, sex, cardiovascular diseases, and risk factors, needs to be included in future TH studies.

## Data availability statement

The original contributions presented in the study are included in the article/**Supplementary Material**, further inquiries can be directed to the corresponding author.

## Ethics statement

The animal study was approved by Landesamt für Natur, Umwelt und Verbraucherschutz Nordrhein-Westfalen, Germany. The study was conducted in accordance with the local legislation and institutional requirements.

## Author contributions

HK: Writing – original draft, Investigation, Formal analysis. JG: Writing – review & editing, Investigation, Formal analysis. SG: Writing – review & editing, Formal analysis. GSH: Writing – review & editing, Investigation, Formal analysis. SD: Writing – review & editing, Formal analysis. JM: Writing – review & editing, Validation. NH: Writing – review & editing, Formal analysis. FK: Writing – review & editing, Validation. LCM: Writing – review & editing, Validation. KL: Writing – original draft, Conceptualization, Investigation. DF: Writing – original draft, Conceptualization.
