# Supplementary material for: Cardiac recovery from pressure overload is not altered by thyroid hormone status in old mice
Source: Front Endocrinol (Lausanne). 2024 Feb 22;15:1339741. doi: 10.3389/fendo.2024.1339741 (PMC10917895; doi:10.3389/fendo.2024.1339741)
Supplement: Supplementary file 1 [file DataSheet_1.docx]

**Supplemental material**


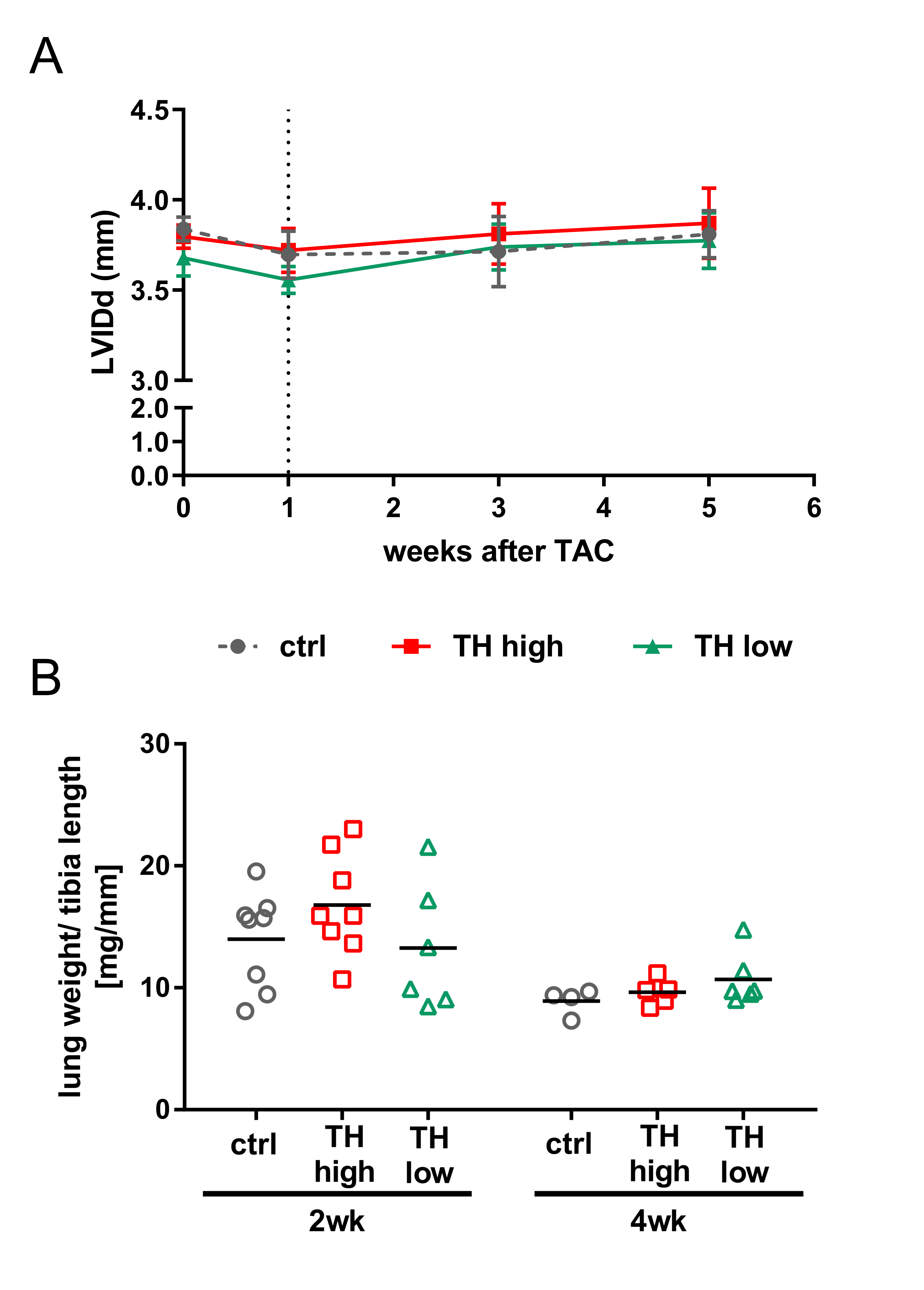


**Suppl. Figure 1:** Left ventricular inner diameter and lung weights of mice subjected to TAC. Diastolic left ventricular inner diameter (LVIDd; A) was quantified by echocardiography during experimental time line prior TAC and 1, 3 and 5 weeks after TAC surgery and lung weight to tibia length ratios 3 or 5 weeks after TAC (i.e. after 2 or 4 weeks of subsequent modulation of TH status by T4 treatment (TH high) or TH deprivation (TH low)) (B). Values are indicated as mean ± SEM or scatter dot plot and mean with no significant changes according to Two-Way ANOVA and Tukey´s *post hoc* analysis, ctrl=control, wk=weeks. Dotted line represents start of T4 or LoI/MMI/ClO_4-_ treatment.


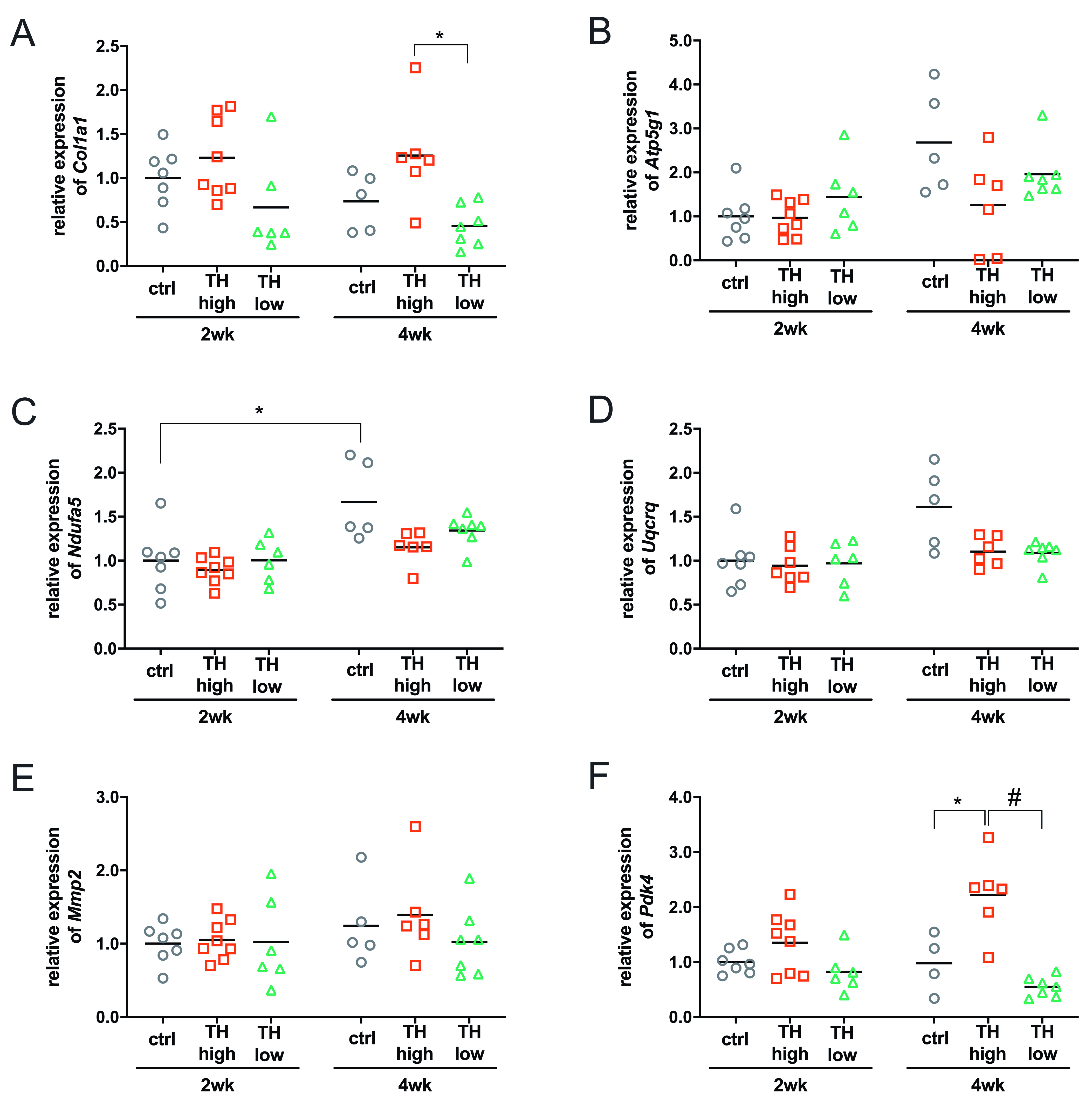


**Suppl. Figure 2:** Expression of genes associated to TAC in hearts of mice subjected to TAC and subsequent modulation of TH status by T4 treatment (TH high) or TH deprivation (TH low). Amount of *Col1a1* (**A**), *Atp5g1* (**B**), *Ndufa5* (**C**), *Uqcrq* (**D**), *Mmp2* (**E**) and *Pdk4* (**F**) transcripts were determined in mouse hearts by qRT-PCR after 2 and 4 weeks of treatment. Scatter dot plot and mean in all panels, One-Way ANOVA with Tukey`s *post-hoc* test, **p*<0.05, ^#^*p*<0.0001, ctrl=control, wk=weeks.


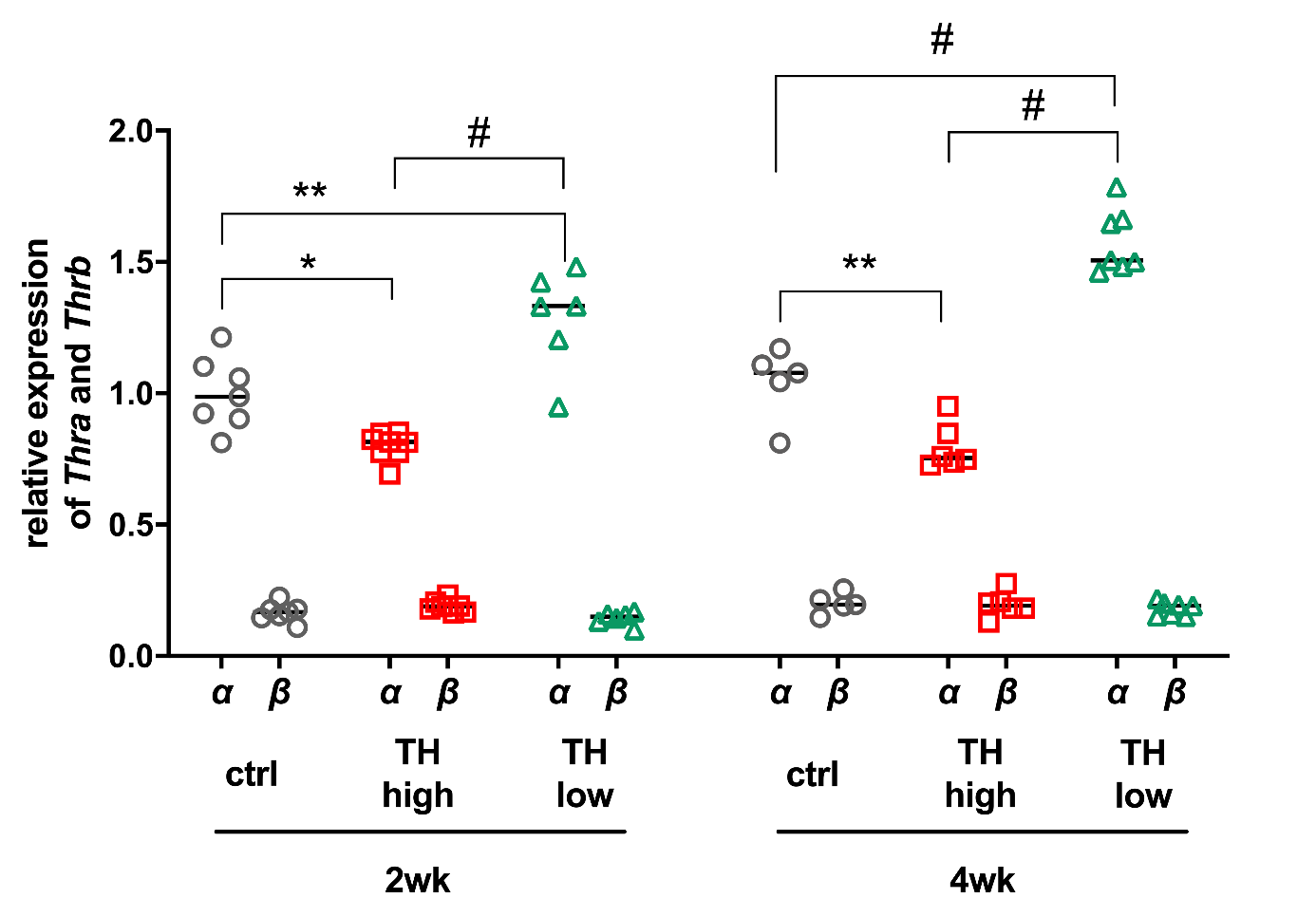


**Suppl. Figure 3:** Expression analysis of TRα and TRβ. Transcripts of total *Thra* and *Thrb* were determined in mouse hearts by qRT-PCR after 2 and 4 weeks of treatment. Scatter dot plot and mean in all panels, One-Way ANOVA with Tukey`s *post-hoc* test, **p*<0.05*,* ***p*<0.01, *#p*<0.0001; ctrl=control, wk=weeks.

**Suppl. Table 1:** Oligonucleotides for quantitative RT-PCR. Oligonucleotides were designed using PrimerBlast (NCBI) and synthesized by Eurofins (Eurofins MWG Synthesis, Ebersberg, Germany).

| Gene name | Forward primer (5’-3’) | Reverse primer (5’-3’) |
| --- | --- | --- |
| *Gapdh* | CCTCGTCCCGTAGACAAAATG | TGAAGGGGTCGTTGATGGC |
| *Rn18S* | CGGCTACCACATCCAAGGAA | GCTGGAATTACCGCGGCT |
| *Polr2a* | CTTTGAGGAAACGGTGGATGTC | TCCCTTCATCGGGTCACTCT |
| *Anp* | TCGTCTTGGCCTTTTGGCTT | GGTGGTCTAGCAGGTTCTTGAAAT |
| *Bnp* | GTTTGGGCTGTAACGCACT | TCACTTCAAAGGTGGTCCCAG |
| *Myh6* | CAGACAGAGATTTCTCCAACCCA | GCCTCTAGGCGTTCCTTCTC |
| *Myh7* | CACGTTTGAGAATCCAAGGCTC | CTCCTTCTCAGACTTCCGCA |
| *Serca2a2* | AACTACCTGGAACAACCCGC | TCATGCAGAGGGCTGGTAGA |
| *Pln* | TTCATGCTCTGCACTGTGACG | GCCAAATGTGAGCTGTCTTCTTTT |
| *Ryr2* | AGGGCAATGAACACTACGGG | CATCCTGCCTACTTGTGCCA |
| *Thra (total)* | GAAAAGCAGCATGTCAGGGTA | GGATTGTGCGGCCAAAGAAG |
| *Thra1* | GCTTCTGACGCCATCTTTGAAC | TCGACTTTCATGTGGAGGAAGC |
| *Thra2* | GCTTCTGACGCCATCTTTGAAC | ATTCCGAGAAGCTGCTGTCC |
| *Thrb* | GGACAAGCACCCATCGTGAA | ACATGGCAGCTCACAAAACAT |
| *Dio2* | GTGACTGGGGAAGCAGAGTG | AGTTTAACCTGTTTGTAGGCATC |
| *Dio3* | GATAGGGAAAGGGTGGGCAG | CTTTAGGCGCTGTTTCGAGC |
| *Col1a1* | CCCTGGTCCCTCTGGAAATG | GGACCTTTGCCCCCTTCTTT |
| *Mmp2* | TCGCCCATCATCAAGTTCCC | CCTTGGGGCAGCCATAGAAA |
| *Atp5g1* | CTAAAGCTGGGAGATTGAAA  AAA | CAGGAAGGCTGCTTAGATGG  TGG |
| *Ndufa5* | GGTCAAGGCGGAGCCAGATG | TCCACTGGTTAGCAGGCGG |
| *Uqcrq* | TCTCCTACAGCTTGTCGCCC | GCTCAAACTCCTGGTTGCC |
| *Pdk4* | TCTGAGGATTACTGACCGCC | CAAAACCAGCCAAAGGGGCA |

| **12 months** | **basal (n=56)** | **1w TAC (n=46)** | **3w TAC control (n=13)** | **3w TAC TH high (n=15)** | **3w TAC TH low (n=15)** | **5w TAC control (n=5)** | **5w TAC TH high (n=7)** | **5w TAC TH low (n=7)** |
| --- | --- | --- | --- | --- | --- | --- | --- | --- |
| **heart rate [bpm]** | 542±65 | 594±78 * | 564±87 | 596±83 | 589±52 | 596±109 | 594±80 | 527±40 |
| **IVSd [mm]** | 0.70±0.05 | 0.98±0.08 * | 1.01±0.08 * | 1.11±0.12 *^,#^ | 0.97±0.10 * | 1.00±0.07 * | 1.02±0.10 * | 0.88±0.08 * |
| **IVSs [mm]** | 1.04±0.10 | 1.25±0.12 * | 1.31±0.12 * | 1.39±0.18 * | 1.27±0.13 * | 1.29±0.06 * | 1.26±0.12 * | 1.24±0.18 * |
| **LVIDd [mm]** | 3.77±0.29 | 3.61±0.43 | 3.71±0.67 | 3.81±0.63 | 3.74±0.48 | 3.81±0.26 | 3.87±0.48 | 3.77±0.38 |
| **LVIDs [mm]** | 2.46±0.28 | 2.62±0.47 | 2.72±0.76 | 2.72±0.80 | 2.68±0.62 | 2.91±0.42 | 2.87±0.54 | 2.75±0.32 |
| **LVPWd [mm]** | 0.71±0.04 | 0.98±0.08 * | 1.01±0.10 * | 1.09±0.10 * | 0.95±0.08 * | 0.99±0.08 * | 1.02±0.09 * | 0.85±0.08 *^,#^ |
| **LVPWs [mm]** | 1.13±0.09 | 1.29±0.11 * | 1.38±0.14 * | 1.44±0.18 * | 1.29±0.12 * | 1.29±0.09 | 1.37±0.13 * | 1.17±0.09 |
| **FS [%]** | 34.87±4.41 | 28.03±5.57 * | 27.83±7.48 * | 29.99±9.24 | 29.15±7.83 * | 23.97±6.82 * | 26.22±6.49 * | 27.09±4.94 * |
| **LV Mass [mg]** | 90.49±11.19 | 133.41±27.59 * | 144.89±31.33 * | 171.48±35.47 * | 136.03±20.71 * | 147.67±16.84 * | 157.89±34.38 * | 118.60±12.40 |
| **LV Mass corrected [mg]** | 72.39±8.95 | 106.73±22.07 * | 115.91±25.06 * | 137.19±28.38 * | 108.82±16.57 * | 118.13±13.47 * | 126.31±27.51 * | 94.88±9.92 |
| **LV Vol d [µl]** | 61.24±11.36 | 56.10±16.43 | 61.64±31.27 | 65.02±26.86 | 61.13±19.82 | 62.78±10.07 | 66.23±19.27 | 61.88±13.11 |
| **LV Vol s [µl]** | 21.84±6.22 | 26.35±12.07 | 31.10±25.22 | 31.53±23.52 | 28.90±18.09 | 33.59±11.33 | 33.28±16.21 | 28.91±7.31 |
| **AV Peak Velocity [mm/s]** | nd | -4539±353 | -4533±297 | -4366±573 | -4806±387 | -4475±532 | -4438±241 | -4420±271 |
| **AV Peak Pressure [mmHg]** | nd | 82.91±12.98 | 82.53±11.10 | 77.25±17.10 | 92.99±14.67 | 81.23±19.29 | 79.01±8.68 | 78.44±9.82 |

**Suppl. Table 2:** Echocardiographic parameters of all time points. Values are represented as mean ± standard deviation, n=number of animals, nd=not determined. **p<0.05* compared to basal values, *#p<0.05* compared to control group at the indicated time (3 or 5 weeks), by One-Way ANOVA and Tukey´s *post hoc* analysis.
